# Epitope-Specific Response of Human Milk Immunoglobulins in COVID-19 Recovered Women

**DOI:** 10.3390/pathogens10060705

**Published:** 2021-06-05

**Authors:** Tatyana V. Bobik, Nikita N. Kostin, George A. Skryabin, Polina N. Tsabai, Maria A. Simonova, Vera D. Knorre, Yuliana A. Mokrushina, Ivan V. Smirnov, Julia A. Kosolapova, Valentina V. Vtorushina, Evgeniya V. Inviyaeva, Evgeniya Polushkina, Ulyana L. Petrova, Anna V. Levadnaya, Lyubov V. Krechetova, Roman G. Shmakov, Gennadiy T. Sukhikh, Alexander G. Gabibov

**Affiliations:** 1 Shemyakin-Ovchinnikov Institute of Bioorganic Chemistry, Russian Academy of Sciences, 117997 Moscow, Russia; bobik_tanya@ibch.ru (T.V.B.); nkostin@ibch.ru (N.N.K.); skryabin@ibch.ru (G.A.S.); polinatsabai@ibch.ru (P.N.T.); simonova@ibch.ru (M.A.S.); vera.knorre@ibch.ru (V.D.K.); yuliana@ibch.ru (Y.A.M.); smirnov@ibch.ru (I.V.S.); 2 Federal State Institution “National Medical Research Center for Obstetrics, Gynecology and Perinatology named after Academician V.I. Kulakov”, Ministry of Health of the Russian Federation, 117997 Moscow, Russia; yu_kosolapova@oparina4.ru (J.A.K.); v_vtorushina@oparina4.ru (V.V.V.); e_inviyaeva@oparina4.ru (E.V.I.); e_polushkina@oparina4.ru (E.P.); u_petrova@oparina4.ru (U.L.P.); a_levadnaya@oparina4.ru (A.V.L.); l_krechetova@oparina4.ru (L.V.K.); r_shmakov@oparina4.ru (R.G.S.); g_sukhikh@oparina4.ru (G.T.S.)

**Keywords:** human milk, breastfeeding, SARS-CoV-2, class A immunoglobulins, class G immunoglobulins, class M immunoglobulins, receptor-binding domain (RBD), S-protein, N-protein, passive immunity

## Abstract

The breastfeeding of infants by mothers who are infected with SARS-CoV-2 has become a dramatic healthcare problem. The WHO recommends that infected women should not abandon breastfeeding; however, there is still the risk of contact transmission. Convalescent donor milk may provide a defense against the aforementioned issue and can eliminate the consequences of artificial feeding. Therefore, it is vital to characterize the epitope-specific immunological landscape of human milk from women who recovered from COVID-19. We carried out a comprehensive ELISA-based analysis of blood serum and human milk from maternity patients who had recovered from COVID-19 at different trimesters of pregnancy. It was found that patients predominantly contained SARS-CoV-2 N-protein-specific immunoglobulins and had manifested the antibodies for all the antigens tested in a protein-specific and time-dependent manner. Women who recovered from COVID-19 at trimester I–II showed a noticeable decrease in the number of milk samples with sIgA specific to the N-protein, linear NTD, and RBD-SD1 epitopes, and showed an increase in samples with RBD conformation-dependent sIgA. S-antigens were found to solely induce a sIgA1 response, whereas N-protein sIgA1 and sIgA2 subclasses were involved in 100% and 33% of cases. Overall, the antibody immunological landscape of convalescent donor milk suggests that it may be a potential defense agent against COVID-19 for infants, conferring them with a passive immunity.

## 1. Introduction

By April 2021, severe acute respiratory syndrome coronavirus 2 (SARS-CoV-2) had affected 147 million patients, and the coronavirus disease 2019 (COVID-19) death toll exceeded 2.5 million worldwide. For many countries, the SARS-CoV-2 pandemic has been a dramatic stress test for their national healthcare systems [1,2]. The issues of childbirth and neonatology during the pandemic have been of particular concern since immunity to SARS-CoV-2 in infants is an essential component for attaining herd immunity [3]. Due to the immaturity of the immune system in infants under 1 year of age, newborns and breastfed babies require special attention during the ever-evolving coronavirus pandemic. In most cases, children with COVID-19 have shown mild symptoms; however, approximately 10% of infants under 1 year of age develop severe symptoms that require special attention, and, in some cases, lead to death [4,5,6].

At present, the SARS-CoV-2 vaccines are not intended for infants [7], which raises the question of how this group is to be protected during the pandemic. For infants, the main source of passive immunity is through maternal milk. In particular, breastfeeding is well known to reduce the probability of death in children under one year of age, and it also reduces the risk of viral infections in infants [8,9]. Despite the WHO recommendations [10] clearly stating the need for breastfeeding of infants by mothers infected with SARS-CoV-2, there has been a significant range of difference between the responses of nations regarding this issue [11]. At the onset of the global COVID-19 pandemic, the lack of understanding of SARS-CoV-2 transmission mechanisms led to the temporary separation of infected mothers from their babies, and the interruption of breastfeeding during the time of the mother’s COVID-19 infection. Currently, the WHO European Office recommends continuing breastfeeding in cases of mild COVID-19 symptoms because even a short delay in breastfeeding initiation can interfere with lactation and increases the risk of infant morbidity and mortality [12,13]. However, the available data on the risk of infection in infants through breastfeeding are extremely limited and contradictory [14,15,16,17,18]. Furthermore, most researchers were unable to prove milk-borne transmission of a live SARS-CoV-2 virus to an infant [11,17,18,19]; however, the risk of contact transmission remains.

Human milk is well known to contain various bio-active compounds, including immunoglobulins, mostly secretory IgA (sIgA) [20,21]. The neutralizing capacity of SARS-CoV-2-specific sIgA, present in convalescent donor milk, may provide an infant with a defense against the disease. Previously, SARS-CoV-2-specific IgG and sIgA were detected in the human milk of infected women [11,22]. Using a broad panel of first-wave patients infected with SARS-CoV-2, we have unambiguously shown that serum IgA are a marker of infection development, and their stable response clearly correlates with the persistence of the virus in the patient’s body [23]. However, analysis of immunoglobulins in the milk of women affected by COVID-19 during pregnancy has only been reported in a few cases, where infection occurred late in the pregnancy [3,19]. Therefore, the presence of SARS-CoV-2-specific immunoglobulins in the human milk of women affected by COVID-19 in early pregnancy, and their epitope specificity, remains unknown. The purpose of this study was to investigate the SARS-CoV-2 epitope-specific antibody landscape of milk from women who recovered from COVID-19. It was of particular interest to study the dynamics of SARS-CoV-2 epitope-specific IgG and sIgA in the milk of women affected by COVID-19 at various trimesters of pregnancy.

For this purpose, we developed a platform for the immunological screening of a cohort of infected mothers, using a number of recombinant SARS-CoV-2 antigens: spike protein (S-protein), its receptor-binding domain (RBD), its RBD-SD1 and NTD subdomains, and the nucleocapsid protein (N-protein). The system included both linear and conformational epitopes of the viral proteins, which was ensured by the expression of the components in both prokaryotic and eukaryotic cells.

## 2. Results

The study involved 41 women who had recovered from a novel coronavirus infection at various trimesters of their pregnancy (Table 1). All women were patients at the Kulakov National Medical Research Center for Obstetrics, Gynecology, and Perinatology between July and October, 2020. The inclusion criteria were as follows: PCR-confirmed coronavirus infection (COVID-19) during pregnancy and lactation in the postpartum period.

**Figure 1:**
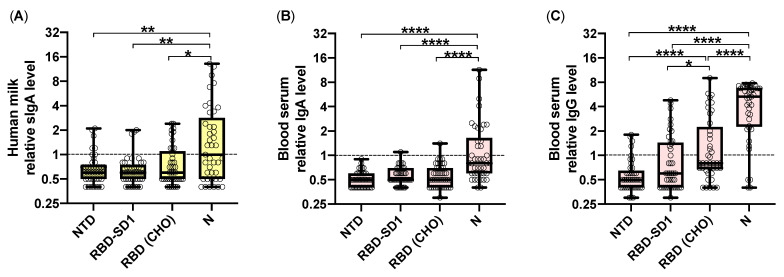
Results of ELISA testing of human milk and blood serum samples of women that recovered from COVID-19 during pregnancy. (**A**) Distribution of the relative antibody levels calculated for IgA (**A**,**B**) and IgG (**C**) specific to SARS-CoV-2 S-protein NTD, RBD-SD1, and RBD (CHO) fragments, and N-protein in human milk (**A**) and blood serum (**B**,**C**). The dashed lines represent the cut-off value, samples with a relative antibody level ≥1 were considered positive. The statistical differences in antigen-specific relative antibody levels were compared between groups by an unpaired, two-tailed Mann–Whitney U-test, significant differences are projected using asterisks (*: *p* < 0.05, **: *p* < 0.01, ****: *p* < 0.0001).

The levels of SARS-CoV-2-specific IgG and IgA in the human milk and blood serum of women who recovered from COVID-19 at various trimesters of pregnancy were determined using an in-house-developed ELISA. As antigens, we used the S-protein RBD-SD1, NTD fragments, and the N-protein produced in the prokaryotic *E. coli* system, adsorbed in a denatured state to plate wells. Recombinant RBD (CHO) produced in the eukaryotic CHO system was used as an antigen representing the conformational RBD epitopes. According to the obtained data (Figure 1A–C), the N-protein-specific sIgA predominated in human milk, and both IgA and IgG were found in the blood serum of the recovered women, which remains consistent with the data previously reported for the serum immunoglobulins [24]. The levels of nucleocapsid-specific antibodies statistically exceed those of conformation-dependent RBD (CHO)-specific immunoglobulins, while the linear NTD and RBD-SD1 epitopes exhibited very low immunogenicity.

An analysis of the blood serum IgG level demonstrated that the immunogenicity of the RBD fragment was largely associated with conformational epitopes. We found no SARS-CoV-2-specific IgG in any of the human milk samples, which is consistent with the available data [11], and is contradictory to other findings [3,19]. A study that demonstrated the lack of SARS-CoV-2-specific IgG in human milk [11] was performed on the milk samples of women who recovered from COVID-19 during the final weeks of pregnancy, and immediately after childbirth. In all likelihood, the particularities of physiological state in pregnant women are the cause of a decrease in the levels of SARS-CoV-2-specific IgG in the blood serum and, therefore, in milk up to undetectable amounts. However, a significantly larger fraction of tested milk samples in some studies [3,19] were obtained from women who were infected several months after childbirth; IgG specific to the S-protein and its fragments were found in human milk in 46% and 80% of cases, respectively. Probably, the milk samples of women who were infected after delivery formed a pool of predominantly IgG-positive samples.

**Figure 2:**
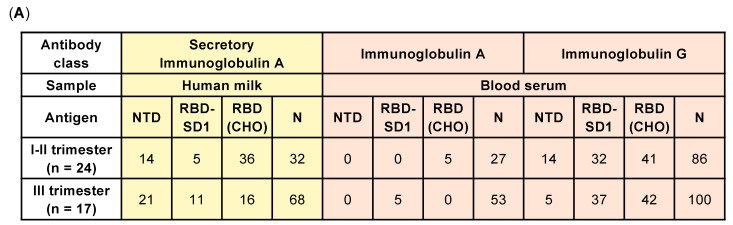

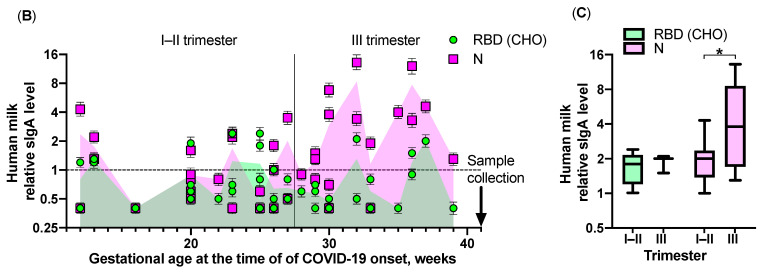
Dependence of the occurrence rate of positive human milk and blood serum samples and relative levels of SARS-CoV-2-specific antibodies at the time of infection. (**A**) Percentage of samples exceeding the cut-off value for an appropriate class of antibodies specific to a specified SARS-CoV-2 antigen. (**B**) Mean individual relative antibody level values of test samples calculated for sIgA specific to SARS-CoV-2 S-protein fragments and N-protein in human milk vs. the gestational age at the time of infection. The dashed lines represent the cut-off value, samples with a relative antibody level ≥1 were considered positive. Error bars represent the SD. (**C**) Distribution of the human milk relative antibody levels of sIgA specific to the SARS-CoV-2 S-protein RBD (CHO) fragment and N-protein. Statistical differences between groups were analyzed using a two-tailed Mann–Whitney U-test (*: *p* < 0.05).

An analysis of the occurrence rate of positive serum and milk samples (Figure 2A,B), within groups of women who recovered from COVID-19 during the first 27 weeks of pregnancy (I–II trimester) and between 28 and 39 weeks (III trimester) revealed a time-dependent change in the rate, which depended on the nature of the antigen.

According to the data obtained, in the group of women infected before the 28th week, the occurrence rate of sIgA in milk samples and IgG in blood serum, specific to N-protein, linear NTD, and RBD-SD1 epitopes in milk, was noticeably reduced. This may be explained by a gradual decrease in the levels of all types of antibodies with a greater amount of time having elapsed before delivery. However, the occurrence rate of conformation-dependent RBD (CHO) sIgA in milk decreased, and the rate of serum IgG did not change. A slight excess (*p* = 0.04, nonparametric Mann–Whitney test) in the levels of N-protein-specific sIgA was found in positive human milk samples in the group of women infected in the third trimester of pregnancy compared to those infected earlier in pregnancy (Figure 2C). However, the levels of RBD (CHO)-specific sIgA in human milk did not differ significantly between the groups, remaining relatively low throughout the study range (12–39 weeks of gestation). An analysis of SARS-CoV-2-specific sIgA subclasses in positive human milk samples revealed that S-antigens solely induce a sIgA1 response, whereas N-protein sIgA1 and sIgA2 subclasses were involved in 100% and 33% of cases, irrespective of trimester groupings.

## 3. Materials and Methods

### 3.1. Development of a Platform for Immunological Screening and Production of Recombinant Proteins and SARS-CoV-2 S- and N-Protein Fragments

Synthesized DNA fragments encoding S-protein RBD-SD1 (330–590 aa) and NTD (17–305 aa) fragments, and the N-protein sequence (1–420 aa) of the SARS-CoV-2 virus were cloned into pET22b plasmids using NdeI and XhoI restriction endonucleases. The accuracy of the produced constructs was confirmed by sequencing. *Escherichia coli* BL21 (DE3) cells transformed with the produced genetic constructs were cultured in a 2YT medium with ampicillin at 37 °C and stirring until OD_600_ = 0.4, with the addition of 1 mM IPTG, and cultured at 30 °C for 6 h. The purification of recombinant proteins from inclusion bodies was performed using metal chelate chromatography (HiTrap FF, GE Healthcare, Uppsala, Sweden) under denaturing conditions. The expression and purification of the recombinant RBD (320–537 aa) produced in eukaryotic CHO cells were performed, as described previously [25,26,27].

### 3.2. ELISA

The produced antigens (100 ng each) in buffer (50 mM sodium bicarbonate, 4 M urea, pH 10.6) were placed into the wells of Nunc MaxiSorp 96-well plates (Nunc, Roskilde, Denmark) and incubated at 4 °C for 16 h. At the end of incubation, a blocking solution (phosphate buffered saline (PBS), 0.1% Tween 20, 3% BSA) was added, and the plates were incubated at 37 °C without stirring for 1 h. After incubation, the blocking solution was removed, and the plates were dried and stored at 6 ± 2 °C. In experiments with biological samples, human milk was centrifuged at 1301 × g and 4 °C for 20 min; the fat-containing layer was discarded, and the supernatant was decanted and diluted at a ratio of 1:10 with PBS, 0.1% Tween 20. Blood sera were diluted at a ratio of 1:10 with PBS, 0.1% Tween 20. Prepared blood serum or human milk samples were placed into the wells and incubated at 37 °C and then stirred for 30 min. Plates were washed 5 times with a washing solution (PBS, 0.1% Tween 20), and the appropriate secondary antibodies were added in a conjugate buffer (PBS, 0.1% Tween 20, 0.1% BSA), incubated at 37 °C and then stirred for 30 min. The following antibodies were employed in this work: anti-human IgG-HRP (Biosan, Novosibirsk, Russia, Cat.# I-3021), anti-human IgA-HRP (Invitrogen, Carlsbad, CA, USA, Cat.# SA1-35467), anti-human IgM-HRP (Biosan, Novosibirsk, Russia, Cat.# I-3022), anti-human IgG1 (HyTest, Turku, Finland, Cat.# 1G2cc), anti-human IgG2 (HyTest, Turku, Finland, Cat.# 1G5), anti-human IgG3 (HyTest, Turku, Finland, Cat.# 1G3cc), anti-human IgG4 (HyTest, Turku, Finland, Cat.# 1G4cc), anti-human IgA1-HRP (Invitrogen, Carlsbad, CA, USA, Cat.# SA5-10198), anti-human IgA2-HRP (Invitrogen, Carlsbad, CA, USA, Cat.# SA5-10199), anti-rabbit IgG-HRP (Sigma–Aldrich, Saint Louis, MO, USA, Cat.# A0545), anti-mouse IgG-HRP (Sigma–Aldrich, Saint Louis, MO, USA, Cat.# A2554). After washing the plates with a washing solution (5 times), the TMB substrate was added and incubated in the dark for 15 min. The reaction was stopped with a 10% phosphoric acid solution, and the absorbance was measured at a wavelength of 450 nm on a plate reader.

All the measurements were performed in triplicate and the values are presented as mean. For the correct interpretation of ELISA, a panel of human milk or blood serum samples from a healthy donor control group was obtained no later than 2018 (*n* = 50). The relative antibody level was calculated as follows: sample signal/(average healthy donor samples signal + 3 SD of healthy donor samples signal). The cut-off value for relative antibody level was chosen as 1 and samples with a relative antibody level ≥1 were considered positive. The data were statistically processed using the GraphPad Prism 8 (San Diego, CA, USA) software. The antibody levels were compared using an unpaired, two-sided Mann–Whitney U-test, *p*-values below 0.05 were considered statistically significant.

## 4. Discussion

To date, vaccination has been considered the most effective strategy for attaining herd immunity to COVID-19. Since the existing vaccines cannot be administered to infants, the protection of this group is of particular importance. On the other hand, in the case of viral infections, the transfusion of convalescent blood [28], and the injection of therapeutic antibodies, particularly for SARS-CoV-2 [25,29,30,31], had demonstrated significant prospects as possible solutions. It is expected that convalescent donor human milk, containing antibodies against SARS-CoV-2, may have similar beneficial effects for babies. Given that it is impossible to prevent the transmission of the virus from an infected mother, the use of convalescent donor milk from mothers who have recovered from COVID-19 to feed virus-negative babies may provide the necessary defenses and will eliminate the consequences of artificial feeding caused by isolation from the child’s biological mother.

It is well known that the virus neutralization capacity of the serum correlates with the level of RBD-specific antibodies [32], and it is possible that human milk with high titers of such antibodies may provide a similar level of protection for infants. In this regard, it was of great interest to characterize the antibody immunological landscape of human milk from women who recovered from COVID-19, especially at the different trimesters of pregnancy.

Our platform for the immunological screening of milk in maternity patients has already provided unambiguous evidence of the presence of conformation-dependent RBD-specific sIgA in a number of human milk samples. 

The protective elements of human milk not only include the neutralizing sIgA antibodies, but also CD8+ T-cells, that were shown to accumulate in newborn’s intestine [33,34]. In addition to the elements of adaptive immunity, the various peptides, lactoferrin, and carbohydrate components will also have a very beneficial impact on the protective functions of the infant and will help to avoid the undesirable consequences caused by artificial feeding. As for future research, an advanced antibody-based proteomics study should be performed to isolate neutralizing IgA [35], and, in parallel, a proper clinical trial is necessary to estimate the outcome of such an approach.

The coronavirus pandemic is, at this time, ongoing, and is entering the phase of a “third wave” in a number of countries. As such, breastfeeding by women who have recovered from SARS-CoV-2 will be the most important factor in the protection of infants through the formation of passive immunity.

## 5. Conclusions

In the milk of women who had had COVID-19 during their pregnancy, we have found a range of sIgA against various SARS-CoV-2 epitopes, such as N-protein; S-protein linear epitopes (NTD, RBD-SD1) and S-protein conformational RBD epitopes. The sIgA against the latter ones were found to be quite persistent, we have detected them even in the milk of mothers who had been affected by COVID-19 during the first trimester of their pregnancy. Based on these findings we may infer that human milk from COVID-19 convalescents possesses a protective effect against SARS-CoV-2 infection.

## Figures and Tables

**Table 1:** Clinical characteristics of women involved in the study.

Indicator	Group 1 (Trimester I of Pregnancy) *n* = 3	Group 2 (Trimester II of Pregnancy) *n* = 21	Group 3 (Trimester III of Pregnancy) *n* = 17	*p*-Value
Age, years (median (min; max))	27 (24; 36)	33 (23; 42)	29 (24; 41)	*p* = 0.159
Mild COVID-19 symptoms, *n* (%)	2 (67%)	16 (76%)	12 (71%)	*p* = 0.779
Moderate COVID-19 symptoms, *n* (%)	0 (0%)	4 (19%)	4 (23%)	*p* = 0.191
Severe COVID-19 symptoms, *n* (%)	1 (33%)	1 (5%)	1 (6%)	*p* = 0.011
Premature birth, *n* (%)	1 (33%) multiple pregnancy	2 (9%) 1—ingrowth of the placenta; 1—intrauterine infection, generally contracted pelvis, 2nd degree	3 (18%) 1—ingrowth of the placenta 2—delivery	*p* = 0.134
Birth at term, *n* (%)	2 (67%)	19 (91%)	14 (82%)	*p* = 0.771
Newborn body weight, g, median (min; max)	2966 (2220; 3878)	3444 (1800; 4222)	3540 (2770; 4340)	*p* = 0.709
Newborn body length, cm, median (min; max)	49.0 (44.0; 55.0)	53.0 (41.0; 58.0)	53.0 (49.0; 56.0)	*p* = 0.300
Apgar score at 1 min after birth, median (min; max)	7.5 (4; 8)	8 (6; 8)	8 (7; 8)	*p* = 0.040
Apgar score at 5 min after birth, median (min; max)	8 (8; 9)	9 (7; 9)	9 (8; 9)	*p* = 0.090

Statistical analysis of clinical data was performed using the MedCalc v16.8 (Ostend, Belgium) software package. To assess the significance of differences, the Kruskal—Wallis method and Fisher's exact test were used. Differences were considered significant at *p* < 0.05.

## Data Availability

The data presented in this study are available on request from the corresponding author. The data are not publicly available due to ethical restrictions.
